# Predicting the Bioconcentration Factor in Fish from Molecular Structures

**DOI:** 10.3390/toxics10100581

**Published:** 2022-09-30

**Authors:** Linda Bertato, Nicola Chirico, Ester Papa

**Affiliations:** Department of Theoretical and Applied Sciences, University of Insubria, 21100 Varese, Italy

**Keywords:** bioconcentration, BCF, QSAR, bioaccumulation, alternatives to animal testing, risk assessment, QSAR-ME Profiler

## Abstract

The bioconcentration factor (BCF) is one of the metrics used to evaluate the potential of a substance to bioaccumulate into aquatic organisms. In this work, linear and non-linear regression QSARs were developed for the prediction of log BCF using different computational approaches, and starting from a large and structurally heterogeneous dataset. The new MLR-OLS and ANN regression models have good fitting with R^2^ values of 0.62 and 0.70, respectively, and comparable external predictivity with R^2^_ext_ 0.64 and 0.65 (RMSE_ext_ of 0.78 and 0.76), respectively. Furthermore, linear and non-linear classification models were developed using the regulatory threshold BCF >2000. A class balanced subset was used to develop classification models which were applied to chemicals not used to create the QSARs. These classification models are characterized by external and internal accuracy up to 84% and 90%, respectively, and sensitivity and specificity up to 90% and 80%, respectively. QSARs presented in this work are validated according to regulatory requirements and their quality is in line with other tools available for the same endpoint and dataset, with the advantage of low complexity and easy application through the software QSAR-ME Profiler. These QSARs can be used as alternatives for, or in combination with, existing models to support bioaccumulation assessment procedures.

## 1. Introduction

The knowledge of chemical properties and of the potential behaviour of chemicals in the environment is an essential requirement in various regulatory frameworks that aim to ensure a high level of protection for human health and the environment through the safe and sustainable use of chemicals [1]. Annex XIII of REACH contains criteria for the identification of persistent (P), bioaccumulative (B) and toxic (T) substances (PBTs); according to these criteria a substance is considered bioaccumulative or very bioaccumulative when the bioconcentration factor (BCF) in aquatic species is higher than 2000 or 5000, respectively [2]. Bioaccumulation is a process whereby a chemical substance is absorbed in an organism by all routes of exposure until a steady state is reached in the organism, i.e., through the diet and the environment [3]. Bioconcentration describes “the process by which a chemical substance is absorbed by an organism from the environment only through its respiratory and dermal surfaces, i.e., chemical exposure in the diet is not included” [3]. The BCF can be calculated as “the ratio of the chemical concentration in the organism and the chemical concentration in the water at steady state, i.e., BCF_SS_ = C_B_/C_WD_” [3]. Bioconcentration in fish is, together with the octanol/water partition coefficient (log Kow), the most used metric to assess chemicals bioaccumulation in risk assessment frameworks all over the world [2]. Furthermore, recent studies have highlighted the need to increase the number of metrics used to describe bioaccumulation, such as those associated with biomagnification (i.e., “the ratio of the chemical concentration in an organism to that in its diet at steady state” [3]) or metabolism [3,4,5,6,7], which are more relevant, for instance, to characterize bioaccumulation in terrestrial organisms.

A big issue associated with the quantification of bioaccumulation metrics is their high experimental cost, time-wise and monetary-wise, as well the large number of vertebrate animals necessary to perform experiments [8,9,10]. For this reason, computational methods such as Quantitative Structure-Activity Relationships (QSARs) have been developed and used for the prediction of bioaccumulation related parameters [4,5,6,7,8,9,10,11,12,13,14,15,16,17,18,19,20,21,22,23,24,25,26,27,28,29]. A recent report by the European Chemicals Agency (ECHA) highlighted that QSARs for the prediction of log BCF are among the in silico approaches that are used more often to replace animal testing within the REACH registration phase [11].

Linear correlations between log BCF and log Kow [12,13,14,15,16,17,18] have been used since the 1980s, to describe and predict the potential bioaccumulation of organic chemicals. However, it is known that the log BCF–log Kow linear relationship only exists within the log Kow range 2–6 and is prone to underestimate the accumulation of substances with non-lipid mediated accumulation mechanisms (e.g., binding to proteins) or to overestimate the accumulation of compounds undergoing biotransformation [3,10].

In the last two decades several models and free tools were proposed to predict log BCF (e.g., [8,9,10,11,12,13,14,15,16,17,18,19,20,21,22,23,24,25,26,27,28,29] EPISUITE [30], VEGA [31], OPERA [32], QSAR DataBank repository [33]) which are based on theoretical molecular descriptors, with or without the inclusion of log Kow, and using a variety of linear and non-linear approaches. Performance of the most widely used QSAR tools available for the prediction of log BCF has been investigated and reviewed recently by other authors [8,9,10,24]. These studies demonstrated a general good quality of the available tools but a large variability in their predictive ability, mostly depending on their applicability domain and on the dimension of the initial training set used to generate the models; for example, the overview given by Lunghini et al. [24] reported a range of *RMSE* values calculated for EPISUITE [30], VEGA [31], and OPERA [32] between 0.43 and 1.57. Furthermore, the analysis proposed by Grisoni [10] highlighted that complex models, i.e., those including several molecular descriptors and properties and based on different modelling approaches, do not necessarily have better performance in prediction compared to simpler models. The study also suggested that the inclusion of descriptors accounting for mechanistic aspects, such as metabolism or protein binding, may be more useful to reduce the error in prediction than increasing the model complexity. This is particularly relevant for specific classes of compounds still underrepresented in the existing experimental dataset such as siloxanes or perfluorinated compounds.

In a recent study, Lunghini and colleagues [24] developed new models based on machine learning techniques (i.e., Support Vector Machines and Random Forest) and a consensus approach. The QSARs were developed starting from a large and curated dataset composed of over 1300 compounds, compiled by merging several log BCF datasets currently available in the literature. These models were developed using ISIDA Property labelled fragment descriptors (from 300 to 5917 depending on the model) [34]. The predictive ability of a consensus model, generated from the combined models, was evaluated on a dataset consisting of chemicals of particular interest for industry. These results were subsequently compared with performances obtained for the same industrial dataset using other existing tools [24]. Lunghini’s work highlighted that the consensus model based on ISIDA descriptors and a heterogeneous structural domain, performed better than most of the used QSAR tools currently available to predict log BCF (linear and non linear), which were based on smaller datasets and applicability domains. Finally, the efficiency of consensus approaches to improve the quality of single models has been highlighted by multiple authors. The evidence that none among the existing models should be singularly considered as the best [9,24], since the quality of predictions is clearly dependent on the AD and range of the response studied in the respective training sets, supports the development of new validated QSARs for the prediction of log BCF using several approaches and descriptors, in order to have more tools available to address different structural and response domains.

In this study, the raw dataset published by Lunghini and collaborators [24] was used to develop new models, based on Multiple Linear Regression (MLR) and classification by Linear Discriminant Analysis (LDA), compliant with regulatory requirements [2,35,36]. Differently from the regression models proposed by Lunghini, which were based on ISIDA fragments, the new models are based on a small number of statistically selected theoretical molecular descriptors calculated with the free software PaDEL-descriptor [37]. Furthermore, in order to avoid issues due to the inclusion of log Kow, for instance overestimation or underestimation of log BCF, as mentioned before [3,10], or uncertainty due to variability of experimental or predicted log Kow in the equations [8,38], this property was excluded from the modelling.

The relevance of the structural features selected in the models by statistical variable subset selection is evaluated and compared with findings in the literature. Furthermore, to evaluate the predictive power of an additional algorithm not yet explored in the literature for the Lunghini’s dataset [24], descriptors selected in MLR and LDA models were used to train Artificial Neural Networks (ANNs) for regression and classification, respectively. The here proposed MLR and LDA models are available for application in the new free software QSAR Multiple Endpoint Profiler (QSAR-ME Profiler), which is freely downloadable at https://dunant.dista.uninsubria.it/qsar/ (accessed on 29 June 2022). These QSARs can be used as alternatives for, or in combination with, existing models to support bioaccumulation assessment procedures.

## 2. Materials and Methods

### 2.1. Data Set and Data Curation

15,371 experimental log BCF (L/kg bdwt) measured for 1551 heterogeneous compounds were taken from the literature [24]. The dataset included heterogeneous chemicals of environmental and toxicological interest, such as biphenyl derivatives, fluorinated compounds, aliphatic hydrocarbons and metal-organic compounds with log BCF ranging from −3.3 to 6.78.

The correspondence between SMILES and CAS, and their correctness were checked, then SMILES were canonicalized using the Open Babel v. 2.4.1 software (San Diego, CA, USA). Subsequently, 11 experimental log BCF were excluded (Table S1) due to wrong or ambiguous SMILES. In addition, 156 inorganic chemicals, metal-organics, salts and ions were excluded from the final dataset (Table S2). Multiple log BCF values available for the same chemical were averaged (Table S3), for a total of 1395 chemicals included in the final dataset. The range of the averaged values was −1.7 to 5.88.

Preliminary MLR models (not shown) were developed to detect outliers. Sixteen outliers were identified (Table S4) and excluded from the final dataset (see Table S5 for the final dataset). *A priori* classes were generated (Table S3) by discretization of log BCF values on the basis of REACH regulatory log BCF > 3.30 threshold [2,9,39,40]. Therefore, based on this cut off, 227 chemicals were ranked as bioaccumulative (B) and 1168 as not bioaccumulative (not-B). However, due to the heavily unbalanced number of chemicals within the B and the not-B classes, a more balanced subset composed in total of 417 compounds was extracted to develop and validate the LDA model. Details on the development of the balanced subset are reported in Section 2.4.

### 2.2. Theoretical Molecular Descriptors

7941 one-dimensional (1D) and two-dimensional (2D) theoretical molecular descriptors, in addition to fingerprints, were calculated by PaDEL-descriptor software v. 2.21 [37] from canonical SMILES. Constant and nearly constant descriptors as well as descriptors found to be correlated pairwise more than 80% and 95%, respectively, were excluded in a pre-reduction step, prior to modelling. The remaining molecular descriptors were then used as input for the Variable Subset Selection (VSS) procedure, applied to generate and select regression [41] and classification [42] models.

### 2.3. Multiple Linear Regression and ANN

Log BCF was first modelled using Multiple Linear Regression by means of Ordinary Least Squares (MLR-OLS, from now on mentioned as MLR for brevity). To further improve the MLR model selected by VSS, a subsequent use of Artificial Neural Networks (ANN) was explored [43,44]. MLR models and the step-up procedure for Variable Subset Selection [41] were developed using in-house written R (version 4.0.2 available from https://cran.r-project.org/ (accessed on 1 July 2020)). This procedure led to a final population of 500 models i.e., 50 models were collected at each size of the step-up procedure, from 1 to 10 variables.

In order to perform external validation, the available chemicals were initially split into a training and a prediction set, where the latter was not used during the calibration of the models. Data were sorted according to increasing experimental response. The first and the last chemicals were for the training set; then, iteratively, the subsequent first chemical was for the prediction set, while the following two were for the training set. Overall, 931 chemicals were put in the training set and 464 in the prediction set. MLR and ANN models were cross validated by a 5-fold CV performed by the R in-house written script. The determination coefficient R^2^ and the Root Mean Squared Error (*RMSE*) were used to measure the models fitting and predictive performances. *RMSE* estimates the error of the model and is calculated as the square root of the average of squared errors in prediction as follows:

$$RMSE=\sqrt{\frac{\sum_{i}{\left(y_{i}-{\hat{y}}_{i}\right)}^{2}}{n}}$$

where n is the number of objects, y_i_ and ŷ_i_ are respectively the experimental and the predicted endpoint values. The advantages of using *RMSE* over other validation metrics were recently highlighted by Consonni and colleagues [45]. *RMSE* was calculated to compare the accuracy of the models when applied to the training (RMSE_Tr_), and the prediction (RMSE_Ext_) sets. *RMSE* was also used to compare the performances of our models with those in the literature. *RMSE* values for the 5-fold CV procedure are reported as RMSE_cv test_. The Y permutation procedure (Y-Scramble) was applied to check the absence of chance correlation between the descriptors and the modelled response, by shuffling the modelled response 50 times, re-developing the models, and averaging the resulting R^2^. Such a metric has been herein referred to as R^2^-YScr, and is expected to be small in robust models (i.e., those with no coincidental relationship between the descriptors and the endpoint), because the QSAR relationship is disrupted by the randomization process. MLR outliers (i.e., compounds with cross-validated standardized residuals greater than 2.5 standard deviation units) and compounds structurally influential in determining model coefficients, were detected graphically by the Williams plot, which displays the Hat values (h) versus standardized residuals. Predictions for high leverage chemicals could be unreliable when they fall outside the structural chemical domain of the training set. Finally, an Artificial Neural Network (ANN) model was generated in KNIME v. 4.2.3. [43] using the RProp MLP Learner [44].

### 2.4. Classification

The BCF dataset listed in Table S5, was used to develop classification models by GA-LDA and ANN. According to the regulatory cut-off log BCF = 3.30, 225 chemicals were identified as potentially bioaccumulative (B), while 1154 as not bioaccumulative (not-B). However, because of the heavily unbalanced number of chemicals included in the two classes, a more balanced subset was created by selecting a similar proportion of B and not-B substances. This selection was performed considering the range of log BCF measured for not-B compounds, i.e., chemicals were sorted by increasing log BCF values and then one every five chemicals was put in the not-B subset. In this way, two *a priori* classes composed of 225 B and 192 not-B compounds were used for classification. The remaining 962 not-B chemicals were used as an additional prediction set.

Linear Discriminant Analysis (LDA) was applied to develop classification models and, to this end, the software QSAR-Co [42] was used. This software allows VSS by means of a Genetic Algorithm. As it was performed for the regression models, chemicals were split according to structural similarity into a training and a prediction set. The Euclidean distance was used in the QSAR-Co software, as the similarity measure, to select 30% of the chemicals for the prediction set. This partitioning led to a training set of 292 chemicals (i.e., 162 B and 130 not-B) and a prediction set of 125 chemicals (i.e., 63 B and 62 not-B). Finally, a priori probabilities for the B and not-B classes were set in LDA as proportional to group sizes.

The classification metrics used to evaluate model quality were the Area Under the Curve (AUC) and Accuracy (or Non Error Rate), Precision, Sensitivity and Specificity quantified as follows:

Accuracy (Ac) = TP + TN/TP + FP + TN + FN

Precision (P) = TP/TP + FP

Sensitivity (Sn) = TP/TP + FN

Specificity (Sp) = TN/TN + FP

TP, TN, FP and FN are the number of true positives, true negatives, false positives and false negatives of each class (in this study, positives are B compounds and negatives are not-B compounds). The applicability domain analysis was performed using the approach of QSAR-Co software [46,47,48], which is able to identify structural and response outliers on the basis of standardization of the molecular descriptors, or on the basis of the *a posteriori* probability calculated for the modelled classes. As was reported for regression, Artificial Neural Network (ANN) models were generated and evaluated in KNIME v. 4.2.3. [43] using the RProp MLP Learner [44].

## 3. Results

### 3.1. MLR-OLS Regression Models for Log BCF

In the first part of this study, we generated 500 QSAR MLR models for log BCF. A first modelling attempt was conducted using 931 chemicals in the training set, which led to a population of models of up to 10 variables using the step-up procedure, selected by choosing R^2^ as the fitness function. The analysis of the best models led to the identification of 16 chemicals (listed in Table S4) falling outside the structural applicability domain of multiple models, which were excluded in the following modelling steps.

A new population of models was trained on the clean dataset (Table S5) using the step-up procedure up to 10 variables. Models’ performances increased until six variables were included. This number of descriptors was finally chosen as the optimal complexity for the models. The ratio objects/number of descriptors was 150, which is fully compliant with the threshold of at least 5 reported in the OECD guidance [35,36]. The best model in the population was the one with the best balance between fitting and cross validation, after considering the applicability domain as well as the distribution of the residuals in prediction. This MLR QSAR is reported as follows (Equation (1)):

log BCF = −1.44 + 0.80 MWC4 + 0.24 SubFPC171 − 0.10 SubFPC295 − 1.19 maxHBd − 0.06 maxdO − 0.51 IC0(1)

n°obj._Tr_ = 920; n°obj._Pred_ = 459; R^2^ = 0.62; Q^2^_loo_ = 0.61; RMSE_Tr_ = 0.80; 5-fold RMSE_cv test_ = 0.81; RMSE_Ext_ = 0.78; R^2^_ext_ = 0.64; R^2^-YScr = 0.02.

The statistics reported above show that the MLR QSAR model fits the training set well and is expected to predict reasonably the activity of new chemicals. The RMSE calculated for the 5-fold cross validation is comparable with the RMSE calculated for the external prediction set, which confirms the generalizability of the model when applied to a large and heterogeneous external dataset. Furthermore, the R^2^-YScr value is very low, which suggests the absence of chance correlation in the model.

**Figure 1:**
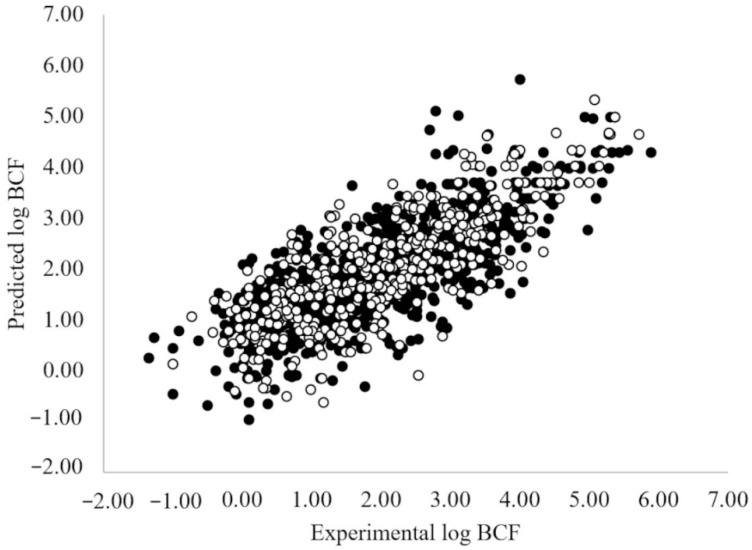
Experimental vs. predicted log BCF values of the best MLR OLS model selected from the step-up population (Equation (1)). Black Dots = training set. White dots = prediction set.

**Figure 2:**
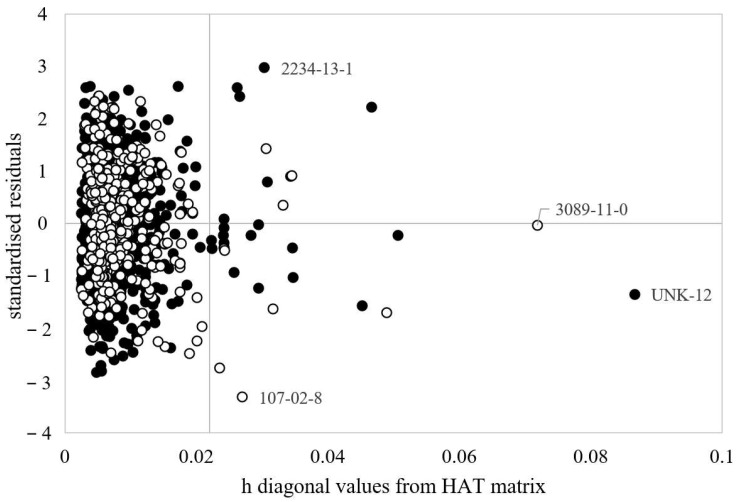
Applicability Domain of the best MLR model selected from the step-up population (Equation (1)). The cut off value on the abscissa for Equation (1) is h* = 0.023. Black Dots = training set. White dots = prediction set.

The plot of experimental vs. predicted values for this model is reported in Figure 1, while Figure 2 shows the applicability domain of the model as described in Section 2.3.

The variables selected in this model are: MWC4, SubFPC171, SubFPC295, maxHBd, maxdO, IC0 [37,49]. MWC4 is the molecular walk count of order 4 (ln(1 + x)); SubFPC171, the second most important variable selected in the model, counts the presence of the SMARTS: [Cl][c], which is the aryl-chloride group. The descriptor SubFPC171 has a positive sign in the equation, meaning that the presence of this fingerprint in the molecular structure increases the value of log BCF. The fingerprint SubFPC295 is related to the presence of heteroatoms and counts the frequency of bonds between C and O, N or S atoms within the molecule. The presence of this fingerprint within the molecular structure decreases the values of log BCF. The descriptor maxHBd is the maximum E-States for (strong) hydrogen bond donors and the maxdO descriptor is the maximum atom-type E-State: =O. Both the e-state descriptors have negative signs in the equation. Finally, the IC0 descriptor is an information content index which brings information related to the symmetry of the molecule.

The applicability domain calculated for the model is reported in Figure 2.

Figure 2 shows that only few chemicals lay far from the central space of the model (i.e., within the horizontal cut off value h* = 0.023) and, in particular, two chemicals are highlighted as heavily out of the AD of the model (i.e., CAS 3089-11-0 and ID UNK-12). These two chemicals are both characterized by large molecular structures reported in Table S6; however, they are well predicted by the model (i.e., value of the standardised residual is close to zero). On the other hand, two additional chemicals, octachloronaphthalene (i.e., CAS 2234-13-1) and acrolein (CAS 107-02-8), are predicted with residuals larger than 3 standard deviations, so they are highlighted as possible response outliers. In addition, they fall outside the applicability domain of the model because of their molecular structure (Table S6).

### 3.2. ANN Regression Models for Log BCF

The six best molecular descriptors selected by the MLR step-up procedure (Equation (1)), and the same training and prediction sets (920 and 459 chemicals respectively), were used to develop Artificial Neural Network (ANN) non-linear models after normalization of the numerical response (log BCF) between zero and one.

**Figure 3:**
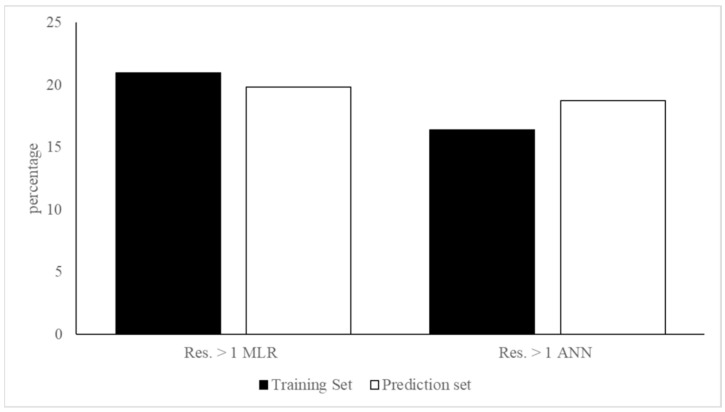
Comparison between MLR and ANN residuals larger than 1 log unit as percentages on the total number of chemicals in the training and in the prediction set.

The ANN model was cross validated by the 5-fold procedure. The best ANN model was chosen by tuning the number of iterations, hidden layers and neurons and considering the fitting and cross-validation performances. The setting for a six variables model was: 1000 iterations, one hidden layer, 10 as the number of neurons (for completeness, the random seed was 10). The statistics of the resulting best ANN model were:

R^2^ = 0.70; R^2^_ext_ = 0.65; RMSE_Tr_ = 0.71; 5-fold RMSE_cv test_ = 0.87; RMSE_Ext_ = 0.76;

These statistics show that the ANN model has good fitting and predictive ability and the RMSE_Tr_ and RMSE_Ext_ values are slightly better than those calculated for the MLR and comparable to the RMSE calculated for the external prediction set. As expected, the variables selected in the MLR approach are still performant when applied with ANN. Figure 3 shows the comparison between MLR and ANN residuals larger than 1 log unit as percentages on the number of chemicals in the training and in the prediction set. Residuals larger than 1 in the training and in the prediction set, as well as the number of common residuals and related percentages, are listed in Tables S7 and S8.

Figure 3 and Table S8 show that ANN is slightly better than MLR having a lower percentage of chemicals with residuals larger than one (16%) compared to the other model (21%), concerning the training set. However, the two approaches have similar performance concerning the prediction set because of comparable percentages of compounds with large residuals (20% and 19% for MLR and ANN, respectively). This behavior reflects the RMSE values calculated for the two models.

Table S8 shows that more than 50% of chemicals with large residuals are shared between the two approaches; therefore, the use of a more complex approach (i.e., ANN) is not helpful to improve the predictivity of the model.

### 3.3. GA-LDA Classification Models for Log BCF

A population of LDA models was generated by the QSAR-Co software using the following settings: number of iterations = 500, size of the GA-population = 100, mutation probability = 0.3, maximum model complexity = 4 variables. Models were cross-validated by means of a 5-fold procedure. The modelled dataset, the values of the molecular descriptors selected in the model and the predictions generated by different classification techniques are reported in Table S9.

The best model within the GA-LDA population was selected considering performance, as well the number of descriptors. Table 1 reports the confusion matrix of LDA which counts the actual and predicted assignments, where the main diagonal contains the correct assignments. Table 2 reports the performance of the model. ROC curves calculated for the GA-LDA model are reported in Supplemental Material Figure S1 A–C.

The model shows good fitting and is suitable for the classification of B and not-B compounds, with comparable accuracy, when it is applied to the prediction set. AUC values are close to or higher than 0.90, thus further supporting the high sensitivity and specificity of the model. ROC curves reported in Figure S1 show the performance of the LDA models in the GA-LDA population.

The variables selected by GA in the best LDA model are: IC2, i.e., the information content index for neighborhood symmetry of second order; TopoPSA, i.e., the topological polar surface area and MAXDP maximum positive intrinsic state difference in the molecule (related to the electrophilicity of the molecule), and MWC4, a molecular walk count representing self-returning counts at length four within the molecule [37,49,50]. It is interesting to highlight that this last descriptor was selected also in the MLR model described in Section 3.1, while MAXDP and TopoPSA were highlighted before in the literature to model log BCF [21,27,29].

### 3.4. ANN Classification Model for Log BCF

The ANN model was cross-validated using the 5-fold procedure. The best ANN model was developed by setting 250 iterations, 1 hidden layer and 20 neurons per layer (random seed was set at 10). The confusion matrix and statistical results are reported below in Table 3 and Table 4 and the ROC curves generated for the ANN model are reported in Figure S2A–C.

As for the GA-LDA, the ANN model has a small percentage of misclassification and the percentages of the remaining quality indicators are mostly above 80%, while AUC is close to 1, so this model is suitable for the classification of B and not-B compounds with comparable accuracy, also when is applied to the prediction set.

### 3.5. Application of the Classification Models to the Not-B Dataset

LDA and ANN classification models were applied to the external dataset excluded from the development of the QSARs, composed of 962 not-B compounds. Performance calculated for each model are reported in Table 5. The dataset and predictions for the 962 not-B compounds are reported in Table S10.

The two models, applied on the very large external dataset, demonstrate good and comparable ability to identify not-B compounds. It is interesting to highlight that the two models misclassify 265 chemicals, where 179 are common misclassifications (Table S11). Furthermore, the range of log BCF for 149 out of the common misclassifications spans from 2 to 3.3, as shown in Figure S3. This suggests that the models mostly misclassify borderline chemicals with log BCF values close to the regulatory cut off. Chemicals that are commonly misclassified within this range belong to classes known as possibly bioaccumulative, such as aromatic organohalogen compounds (e.g., PCBs, dioxins and furans) as well as PAHs and some perfluorinated compounds. This highlights that predictions may be more uncertain for this log BCF range. Possible uncertainties associated with the variability of experimental log BCF as related to regulatory cut off have been highlighted before [51]. Finally, we wish to highlight that, among the common misclassifications, only Hexchloroethane (CAS: 67-72-1) and Hexabromobenzene (CAS: 87-82-1) fall outside the applicability domain identified by the model (QSAR-Co indication).

## 4. Discussion

In this paper we have presented regression and classification models for the prediction of the log BCF of heterogeneous organic chemicals in fish. All the models have been developed and validated considering their statistical quality as discussed below. The regression models were generated using the step-up variable selection procedure [41] which led to the development of MLR and ANN QSARs, based on six descriptors of the molecular structure. These new models are statistically performant considering the internal robustness and predictivity (R^2^ 0.62–0.70, RMSE_cv test_ 0.81–0.87 (5-fold cv), RMSE_Ext_: 0.76–0.78 and R^2^_ext_ 0.64–0.69). These performances are in line with the quality of other literature models developed for the same endpoint but for different datasets [8,9,24,30,31,32]. In addition, the quality of the new regression models is comparable to the quality reported in the literature for the same dataset [24] i.e., R^2^: 0.72–0.75, RMSE_cv test_: 0.68–0.78 (3-fold cv), RMSE_Ext_: 0.77–0.92 and R^2^_ext_ 0.66–0.77, making our new QSARs equally valid for application as the existing models. We would also like to highlight that the approach proposed in the literature is based on a much large number of ISIDA fragments (300–5917) than in our study (six molecular descriptors); moreover, the best performances of the ISIDA models were obtained using a consensus approach based on predictions generated using multiple approaches (i.e., Random Forest (RF) and Support Vector Machines (SVM)). Furthermore, the models proposed here are validated using an external prediction set of 459 chemicals, which is twice the size of the one used to validate ISIDA-based QSARs [24]. Therefore, the new results reported in this paper are satisfactory, in particular considering the small number of descriptors included in the models, which makes them more easily interpretable and applicable, and improves their usefulness in a regulatory context and for risk assessment purposes.

Among the molecular descriptors selected in the MLR model reported in Equation (1), those related to polarizabilities, and thus hydrophobicity, and hydrogen bonding, have been confirmed as very influential in the mechanisms of bioconcentration [10,12,20,21,23,27,28,29]. In more detail: MaxHBd relates to the presence of hydrogen bond donors. SubFPC295 refers to the presence of heteroatoms and counts the frequency of bonds between C and O, N or S atoms within the molecule; while the fingerprint SubFPC171 is associated with the presence of chlorine atoms and its presence increases log BCF. It is interesting to highlight that the result for this last feature is consistent with observations by multiple authors [9,10,21,22,23] as related to an increase of the log BCF value. As expected, in our setup, ANN fits slightly better than MLR does; however, the application of the two models to the external dataset provided similar results. The similar predictivity supports the relevance of the structural features used in these models to predict the log BCF of new chemicals, independently of the algorithm used to generate predictions.

Classification models developed in this study have good fitting and predictivity. A simple LDA model was created using only four molecular descriptors selected with a procedure based on a genetic algorithm (GA). Among the descriptors selected in the model, the topological polar surface area calculated using N, O, S and P atoms (TopoPSA), was reported in the literature by Papa and collaborators [21] and by Kobayashi and Yoshida [29] as particularly relevant to modelling log BCF, as well as MAXDP [21,27].

The application of the GA-LDA classification model, as well as of the ANN classification model, which were calibrated using the four molecular descriptors selected in LDA, successfully predicted the log BCF of an external set composed of 962 not-B compounds. The analysis of common errors generated by the two models highlighted that most of them are in the region of log BCF close to the cut off value of 3.30, which is characterized by higher uncertainty in prediction, requiring an increased attention for possible implications for the models’ results. In particular, chemicals belonging to this region (i.e., PAH, dioxins and other halogenated compounds) have molecular structures typically associated with B chemicals and are overestimated to the B class. Even though the overestimation of a not-B compound to the B class may be considered as more precautionary compared to the underestimation of possible B behaviour, these errors close to the cut off value of 3.30 may be associated with experimental uncertainty. The log BCF uncertainty in the regulatory cut off regions, possibly undermining the outcome of B-assessment, was recently highlighted by Wassenaar and colleagues [51]. Other authors suggested the use of a safety margin at the log BCF = 3.3 threshold as a possible way to accommodate uncertainty in prediction, in this area of the log BCF range [8].

## 5. Conclusions

In this work, we have proposed two QSAR models of regression and classification for the prediction of the regulatory endpoint bioconcentration factor (BCF). These models were created following the OECD guidance for QSAR models development and validation. To the best of our knowledge the data used for the development of the models represent the most recent and the largest curated dataset currently available for this endpoint.

The performance of the new models is in line with already existing tools; therefore, they can be proposed as alternatives or additional tools to increase the use of consensus approaches based on multiple algorithms and descriptors. It should be noted that Lunghini et al. [24] demonstrated that consensus models, based on ISIDA Fragments and trained on log BCF data used to create the here-proposed models, were characterized by a wider applicability domain than the existing tools available for the prediction of log BCF. Future evaluations may be useful to further investigate the applicability domain of the here-proposed QSARs in direct comparison with the other tools, i.e., on the same external prediction sets. Furthermore, since the quality of predictions can be at most as good as the quality of the experimental data, lowering the experimental uncertainty of input data around the regulatory cut off (i.e., log BCF 3.30) could reduce models’ uncertainty in this region of the log BCF range.

The variable subset selection procedure used to create the QSARs led to the selection of a group of nine molecular descriptors which are particularly important for the modelling of the BCF, consistent with similar results described by other models in the literature. However, the new models are based on much simpler algorithms and, compared to other existing tools, are extremely parsimonious since based on only six and four descriptors, respectively, which can be easily calculated using the freely downloadable PaDEL-descriptor software [37]. The reduced complexity of these models also aids in the further uptake of QSAR model results in a regulatory context, since the interpretability of the models’ results becomes more straightforward, enhancing the trust in in silico, non-animal, methods.

Finally, to ease the application of the here-proposed MLR and LDA models to support regulatory decision making in the context of bioaccumulation assessment, and their use as individual tools or in combination with other models, they are implemented in the QSAR-ME Profiler tool, freely downloadable at https://dunant.dista.uninsubria.it/qsar/ (accessed on 29 June 2022).

## Figures and Tables

**Table 1:** Confusion matrix (i.e., percentages of correct assignations as well as misclassifications) for the GA-LDA classification model. True classes are reported in columns, assigned classes are reported in rows.

Training	B	Not-B	Prediction	B	Not-B
B	88	12	B	90	10
not-B	15	85	not-B	23	77

**Table 2:** Quality indicators calculated for the training set and for the prediction set.

	Split	Ac %	P %	Sn %	Sp %	AUC	AUC_cv test_
LDA	Training	87	88	88	85	0.92	0.89
	Prediction	84	80	90	77	0.90	-

**Table 3:** Confusion matrix (i.e., percentages of correct assignations as well as misclassifications) for the ANN classification model. True classes are reported in columns, assigned classes are reported in rows.

	Training	B	Not-B	Prediction	B	Not-B
ANN	B	90	10	B	89	11
	not-B	11	89	not-B	23	77

**Table 4:** Quality indicators calculated for the training set and for the prediction set.

	Split	Ac %	P %	Sn %	Sp %	AUC	AUC_cv test_
ANN	Training	90	91	90	89	0.95	0.92
	Prediction	83	80	89	77	0.88	-

**Table 5:** Number of correct classifications and misclassifications of the 962 not-B molecules predicted by LDA and ANN. In brackets are reported percentages over the 962 B molecules.

	LDA	ANN
Number of correct predictions (%)	730 (76%)	750 (78%)
Number of common misclassifications (%)	179 (17%)	

## Data Availability

Data used to generate the models proposed in this work are available as Supplementary Material. The original raw dataset is published in reference [24].

## Supplementary Materials

The following supporting information can be downloaded at: https://www.mdpi.com/article/10.3390/toxics10100581/s1, Figures S1–S3 Supplementary Material_A; Tables S1–S11: Supplementary Material_B. The Supplementary Files are provided to support the results and the analysis proposed in the manuscript.
