# Supplementary material for: Predicting the Bioconcentration Factor in Fish from Molecular Structures
Source: Toxics. 2022 Sep 30;10(10):581. doi: 10.3390/toxics10100581 (PMC9610932; doi:10.3390/toxics10100581)

Supplementary material for the article:

Predicting the Bioconcentration Factor in Fish from Molecular Structures

Linda Bertato, Nicola Chirico and Ester Papa *

Figure S1 A - ROC curve for the LDA model calculated for the training set

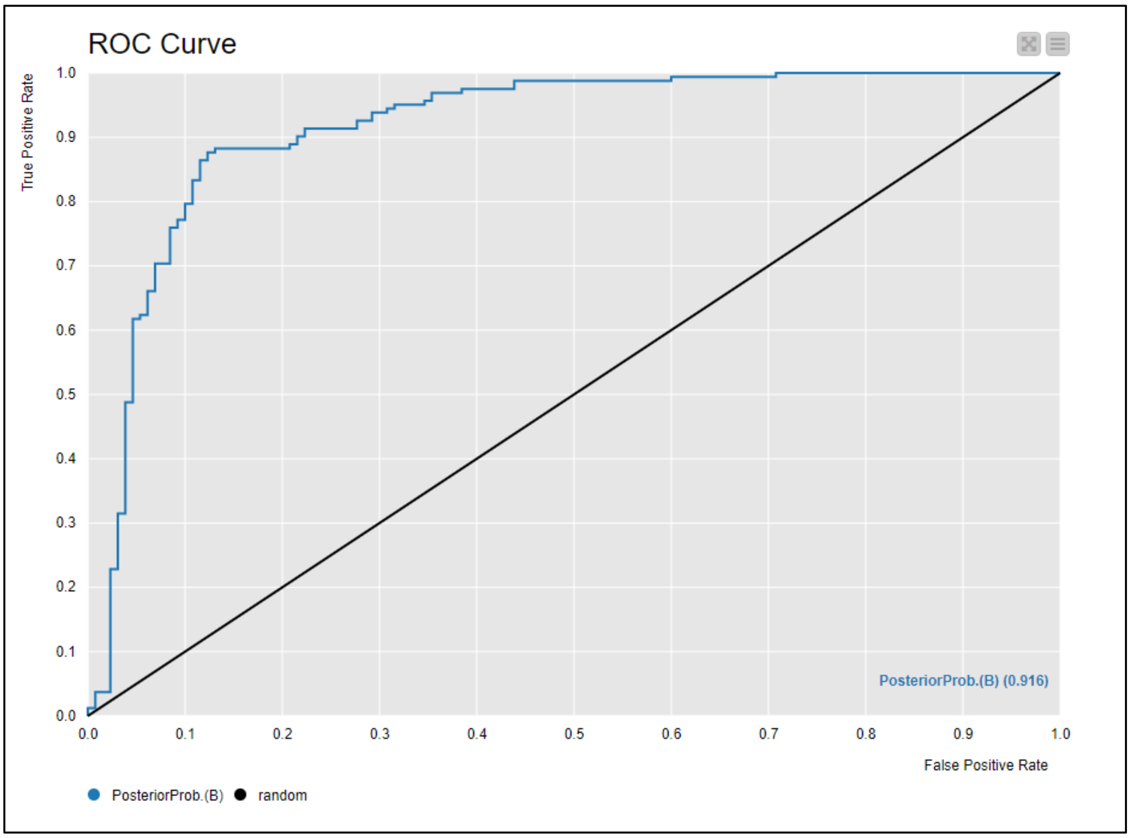

Figure S1 B – ROC curve for the LDA model calculated for the prediction set

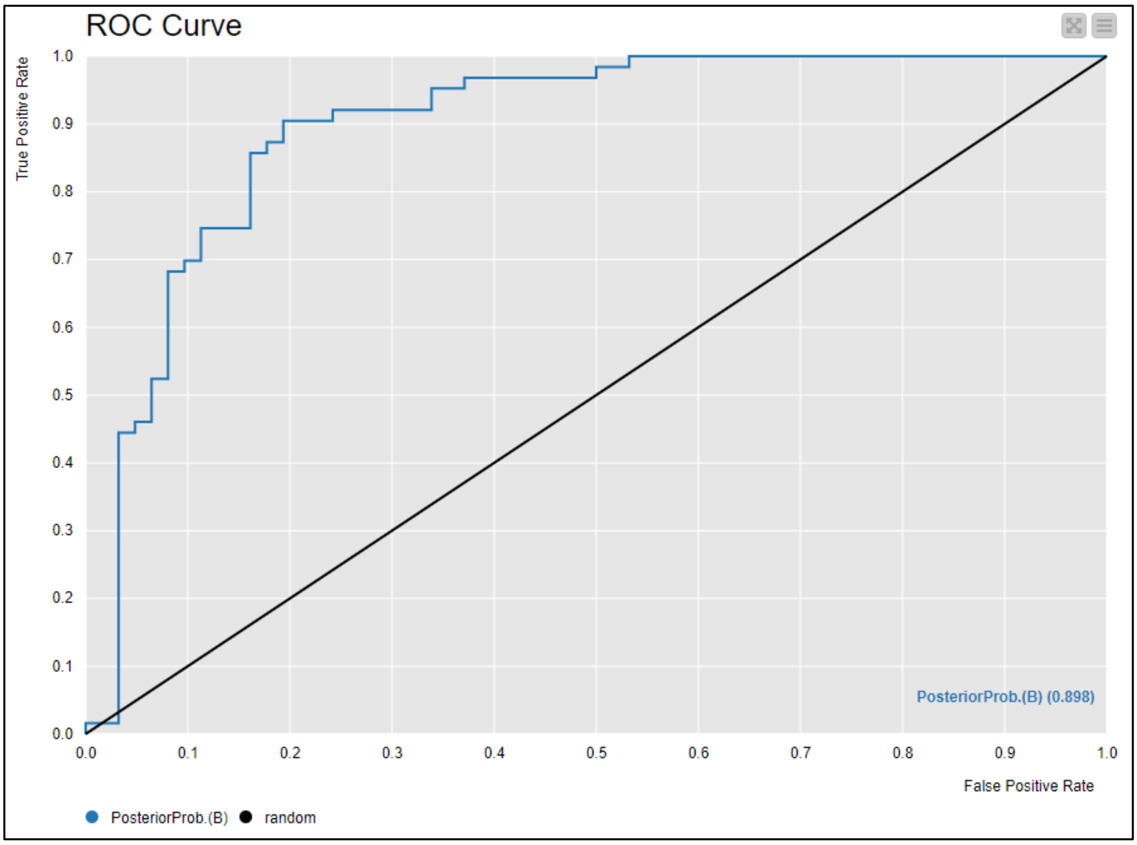

Figure S1 C - ROC curve for the LDA model calculated for the 5-folds cross validation

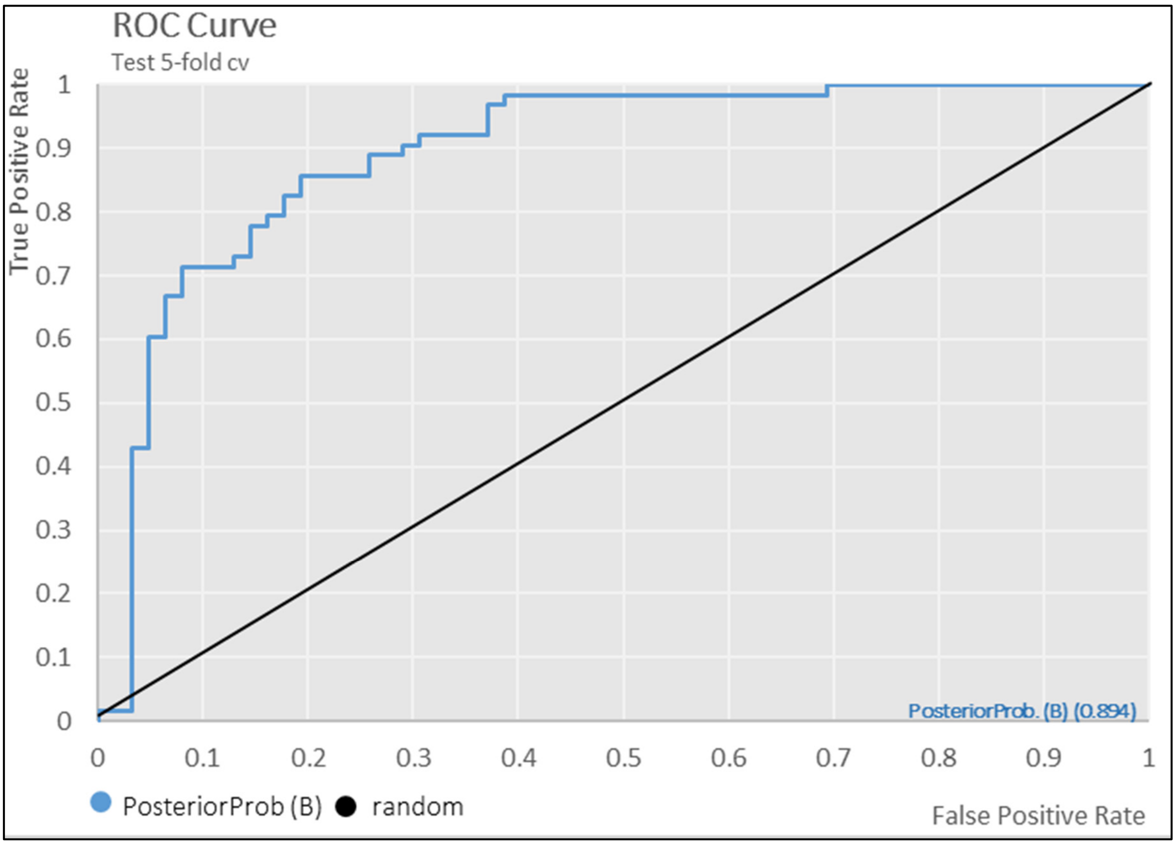

Figure S2 A - ROC curve for the ANN model calculated for the training set

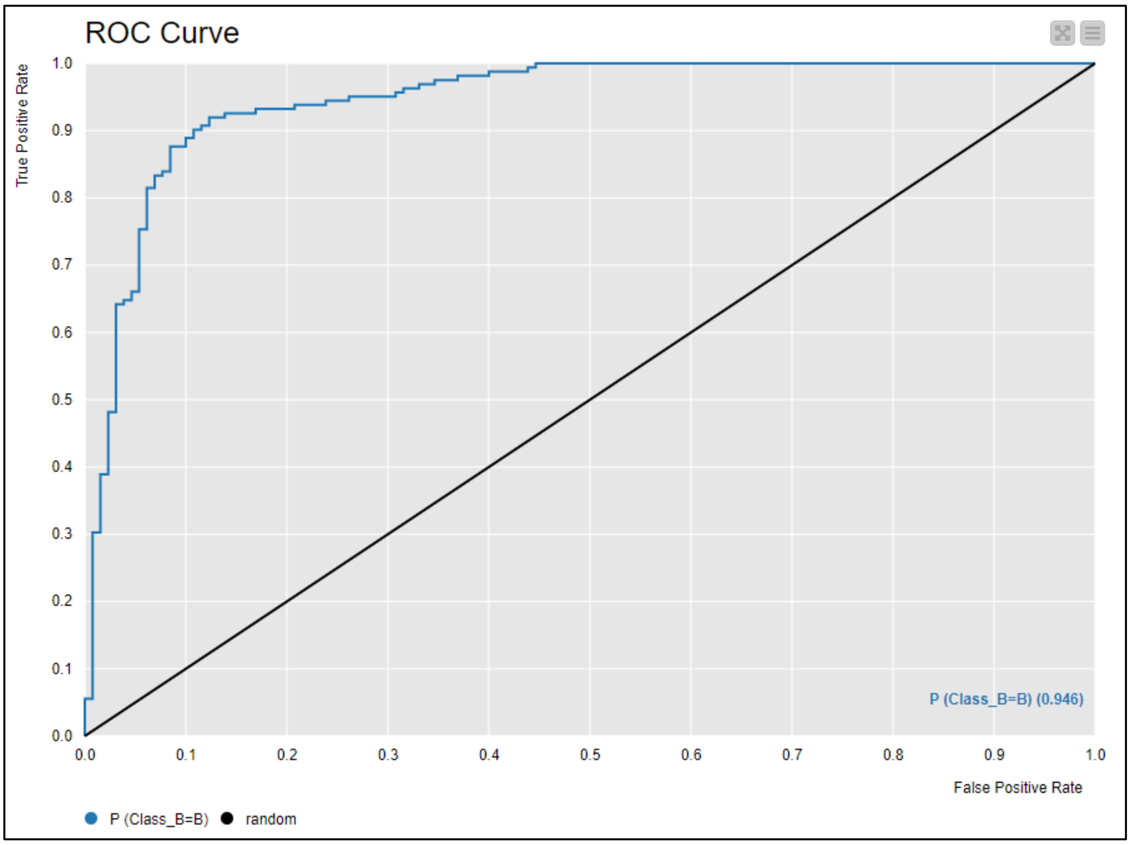

Figure S2 B – ROC curve for the LDA model calculated for the prediction set

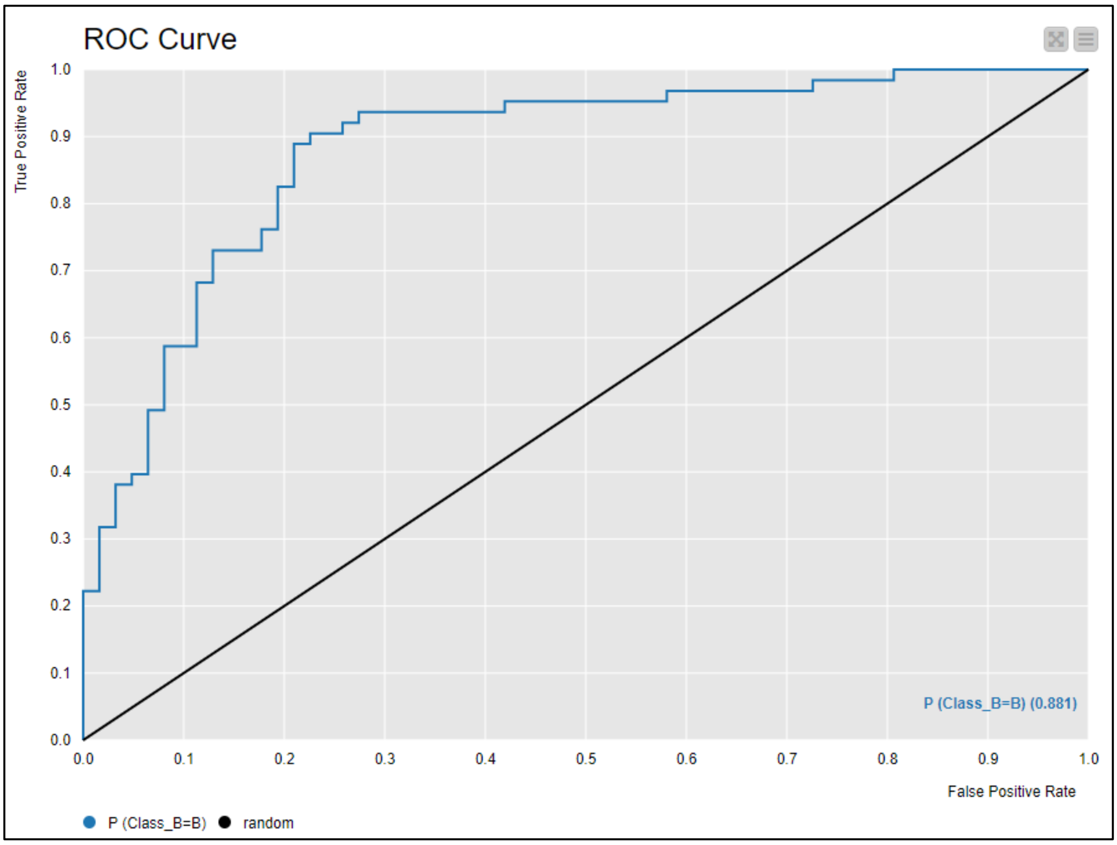

Figure S2 C - ROC curve for the LDA model calculated for the 5-folds cross validation

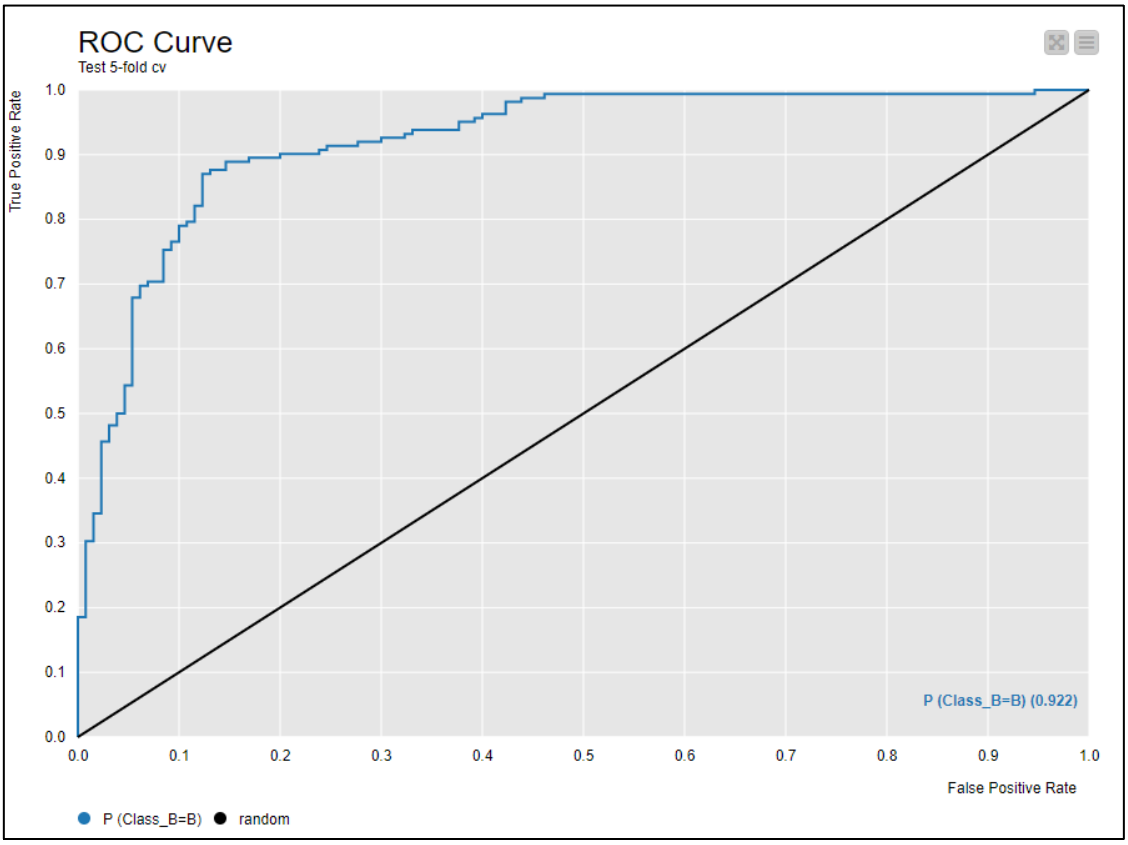

Figure S3. Box plots comparing the experimental range in the external prediction set composed of notB compounds and the distribution of the common errors in prediction among LDA and ANN models

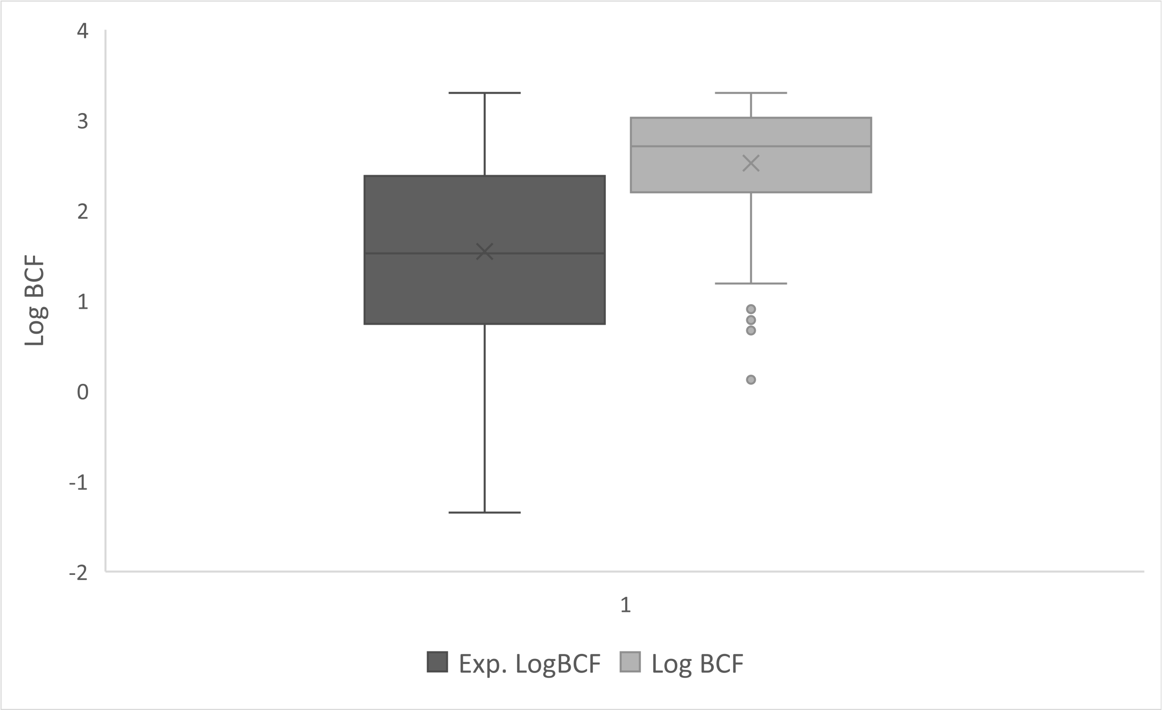

Supplement: Supplementary file 1 [file toxics-10-00581-s001.zip › Supplementary_Material_A.pdf]
